# Differences in Incidence of Acute Viral Hepatitis between Foreigners and Autochthonous Population in Italy

**DOI:** 10.3390/ijerph18157944

**Published:** 2021-07-27

**Authors:** Franca D’Angelo, Luigina Ferrigno, Annamaria Mele, Valeria Alfonsi, Silvia Declich, Giulia De Ponte, Simonetta Crateri, Alessandra Burgio, Susanna Caminada, Maria Elena Tosti, on behalf of The SEIEVA Collaborating Group

**Affiliations:** 1 National Center for Global Health, National Institute of Health, 00161 Rome, Italy; franca.dangelo@iss.it (F.D.); luigina.ferrigno@iss.it (L.F.); silvia.declich@iss.it (S.D.); giulia.deponte73@gmail.com (G.D.P.); simonetta.crateri@iss.it (S.C.); 2 Department of Public Health and Infectious Diseases, University “La Sapienza”, 00185 Rome, Italy; annamaria.mele@uniroma1.it (A.M.); susanna.caminada@uniroma1.it (S.C.); 3 Health Management, Sant’Andrea Hospital, 00189 Rome, Italy; valfonsi@ospedalesantandrea.it; 4 Health Unit, National Institute of Statistics (ISTAT), 00184 Rome, Italy; burgio@istat.it

**Keywords:** acute viral hepatitis, incidence, foreign citizens, Italy

## Abstract

Background: In European countries, the prevalence of HBV and HCV in refugees and migrants tends to reflect the prevalence in their countries of origin. The aim of this study is to analyse acute viral hepatitis cases diagnosed in Italy among foreign citizens and to compare incidence rates in foreigners and Italians. Methods: We analysed the cases of each viral hepatitis type among foreigners. Standardised incidence rates were compared between natives and foreigners. Results: Between 2004 and 2019, 15,872 cases of acute viral hepatitis were notified by 10 Italian regions, 14.8% among foreign citizens. Until 2012, the percentage increased gradually, while a fluctuating trend set in from 2013 onwards; in 2019, 23.9% of cases were foreigners. Data from the SEIEVA surveillance show higher standardised incidence rates of hepatitis A and B among foreign citizens; no significant difference emerged between Italians and foreigners in terms of their hepatitis C incidence. Conclusions: foreign citizens have an increased incidence of hepatitis A and B. Regarding hepatitis A, vaccination is strongly recommended to foreigners travelling to their countries of origin. Screening tests for hepatitis B and C infection should be offered to newly arrived migrants from high prevalence countries, or having specific risk factors.

## 1. Introduction

Migration is a phenomenon of population dynamics, driven by socio-economic, political and environmental factors. Worldwide, the number of migrants tripled since the 1970s reaching 272 million in 2019 [1], with one-quarter being forced migrants [2,3].

The Italian Institute of Statistics (ISTAT) recorded 5,039,637 foreign residents in Italy at 1st January 2020, i.e., 8.4% of the total resident population [4]. These estimates do not represent the number of foreigners present in the country, as they do not include undocumented migrants: the Italian Initiative and Study on Multiethnicity (ISMU) estimates that 562,000 undocumented migrants lived in Italy in 2019 [5]. Foreign citizens present in Italy are prevalently coming from Eastern Europe, Sub-Saharan Area, and Central and Eastern Asia [6]. At the end of June 2020, 84,400 newly arrived migrants and asylum seekers were estimated to be present in Italian reception centres [6].

Viral hepatitis infections are the most common cause of liver diseases and continue to constitute a global public health challenge. The different viruses are present worldwide, but their spread varies from country to country, and it is mainly related to sanitary, economic and environmental conditions [7]. According to a systematic review by the European Centre for Disease Prevention and Control (ECDC) [8] and to a World Health Organization (WHO) Report [9], prevalence of hepatitis B virus (HBV) and hepatitis C virus (HCV) infections among refugees and migrants in the European Region tends to reflect the prevalence of infection at their places of origin: HBV prevalence is reported to be more than 10% among migrants from East Asia, the Pacific and Central, Western and Southern Africa; 4–6% among those from central and South Asia, Eastern Europe and Eastern Africa, and less than 2% among those from North Africa, the Caribbean, Latin America and the Middle East [9,10]. The prevalence of HCV infection is highest among refugees and migrants from sub-Saharan Africa, Asia and Eastern Europe [9]. Studies carried out among migrants and refugees in different Italian cities show prevalence rates of HBV and HCV ranging from 9.6 to 12.2 [11,12,13] and from 3.3 to 6.2 [11,12,14], respectively.

Regarding the epidemiology of HBV and HCV in Italy, the scientific literature reports only local studies on their prevalence among the general population or data restricted to particular groups. In 2018, ECDC published a literature review indicating a range of 0.5–5.8% for HBV prevalence and 0.6–27.6% for HCV prevalence in the general population [15]. A more recent study conducted in 5 metropolitan areas found a 1.7% prevalence of active HCV infection (HCV-RNA positive) [16], while two other studies carried out on cohorts of pregnant women reported prevalence rates of 0.2–0.4% for HCV [17,18] and of 0.5% for HBV [17]. The current risk of acquiring infections in Italy is very low, since the circulation of hepatitis viruses progressively decreased in the last 30 years: the impact of B and C viruses, in particular, was reduced, with an incidence of acute infections of 0.4 and 0.1 on 100,000 population, respectively, in 2019 [19]. The HBV vaccination program in place in Italy since 1991 has certainly contributed to the observed trend [20].

In light of a decreased circulation of hepatitis viruses in Italy, the concurrent increase of the migrant population, often from countries with high and intermediate endemic level of hepatitis [8], makes epidemiological surveillance even more necessary: not only to study the spread of these viruses, including among foreign citizens, but also to identify possible areas of intervention in the interest of public health.

Viral hepatitis infections are subject to mandatory notification by the Italian Ministry of Health. Since 1985, an enhanced voluntary surveillance system, SEIEVA (acronym for Integrated Epidemiological System for Acute Viral Hepatitis), is active within the Italian National Institute of Health, with a mandate to support the mandatory system while investigating further epidemiological aspects [21].

The aim of this study is to analyse acute viral hepatitis cases diagnosed in Italy among foreign citizens and to compare standardised incidence rates among foreign and Italian populations, based on SEIEVA case reports.

## 2. Materials and Methods

The present study is a report from a routine surveillance system active in Italy. It was performed with data from the SEIEVA surveillance, collected from a network of Local Health Units located throughout Italy and covering now more than 82% of the population. An acute hepatitis case is defined as a person with an acute illness compatible with hepatitis and a significant (greater than ten-fold) increase of serum alanine transferase (ALT). Serological criteria used to distinguish hepatitis types are:

- acute hepatitis A: positive hepatitis A virus (HAV) specific IgM antibodies, regardless of other viral markers;
- acute hepatitis B: positive IgM anti-HBc and negative IgM anti-HAV, regardless of other viral markers;
- acute hepatitis C: negative IgM anti-HAV and IgM anti-HBc and positive anti-HCV or HCV-RNA;
- acute nonA-nonC: negative IgM anti-HAV, IgM anti-HBc, anti-HCV/HCV-RNA; IgM anti-HDV (hepatitis Delta virus) and IgM anti-HEV assays were used to further discriminate within nonA-nonC hepatitis cases;
- Unknown: a differential diagnosis was not possible due to missing/inconsistent data on hepatitis markers.

The diagnoses of hepatitis were made in different health facilities throughout Italy, each of which can use different serological tests, always validated.

Demographic, clinical, epidemiological and laboratory data, as well as information on risk factors are collected by a standardised questionnaire, after obtaining informed consent. Since 2004, the SEIEVA surveillance system routinely collects information on the citizenship of cases, making it therefore possible to analyse the occurrence of acute viral hepatitis among foreign citizens. The definition of foreign is based on the information on citizenship (not Italian) collected with the epidemiological questionnaire, regardless of the time spent in Italy.

The study population was constituted by cases notified to SEIEVA by 10 regions participating with all their Local Health Units in the SEIEVA surveillance during the entire period 2004–2019 (4 Regions in Northern Italy, 5 in Central Italy and 1 in Southern Italy). An analysis of cases reporting foreign citizenship was performed for each hepatitis type, also considering a distinction between Strong Migratory Pressure Countries (SMPC) and High-Income Countries (HIC) [22,23].

Age-standardised incidence rates were computed separately for Italian residents and for foreign citizens from SMPC; this was done only for hepatitis A, B and C cases, as nonA-nonC or unknown hepatitis cases do not form a homogenous group [24]. Cases from HIC were excluded from this comparison, as they were observed in small numbers, making rate estimates unstable. The data source for the resident population by citizenship (used as denominator for incidence rates in the selected Regions) is the Italian permanent census: a new data flow was started in 2018 and is based on the integration of administrative and sample surveys’ data; it provides annual figures on the main socio-economic characteristics of the resident population, including data by citizenship, gender, age and municipality of residence [4]. In order to standardize the rates by sex and age, the Italian population residing in the selected regions in 2011 was used as standard population. The rate ratio test was used to test differences between Poisson incidence rates.

## 3. Results

Between 2004 and 2019, 15,872 cases of acute viral hepatitis were notified to SEIEVA by the 10 Italian regions considered. Among these cases, 14.8% (*n* = 2352) were diagnosed among foreign citizens from both SMPC and HIC. The percentage gradually increased over the years until 2012 (from 11.0% to 22.6%), while a fluctuating trend set in from 2013 onwards; in 2019, foreign citizens accounted for 23.9% of cases. The percentage also varies according to the different types of hepatitis: the highest data were observed for nonA-nonC/unknown hepatitis (22.2%), while the percentages of cases having foreign citizenship were respectively 13.3% and 17.0% for hepatitis A and B (Table 1).

Overall, most of the acute viral hepatitis cases among foreign citizens were observed in persons from SMPC (96.9%), while only 74 cases were diagnosed among foreigners from HIC. Most of the notified cases are from Eastern Europe (37.7%), especially hepatitis B and C cases, and Africa (32.9%), especially hepatitis A cases. About 49% of cases nonA-nonC or with unknown aetiology came from Asia (Table 2).

Information concerning the period during which foreign citizens lived in Italy was collected by SEIEVA surveillance since 2009, i.e., for 691 cases: 86.4% of those cases lived in Italy for more than 1 year (median 6 years).

### 3.1. Hepatitis A

During the observation period, 8408 cases of acute hepatitis A were notified to SEIEVA; 1116 (13.3%) of them were foreign citizens. Almost half of these cases were from Africa, including 444 cases from Morocco. Fifty-five cases concerned people from HIC (74% of the total acute viral hepatitis cases diagnosed) (Table 1 and Table 2).

**Figure 1:**
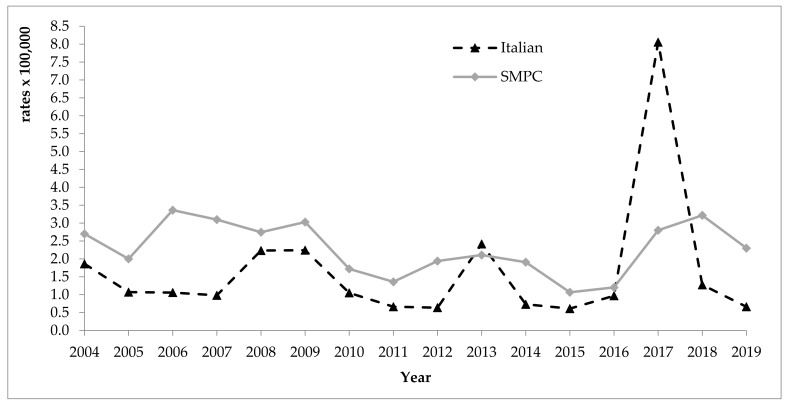
Incidence rates (standardised per 100,000) of viral acute hepatitis A in the foreign population from SMPC and among Italians, years 2004–2019. SMPC—Strong Migratory Pressure Countries.

Figure 1 shows a comparison between the 2004–2019 incidence rates in Italian and in foreign populations from SMPC. We can observe the large 2017 epidemic which affected, in particular, Italian male homosexuals [25], although a slighter increase in incidence rates can also be observed among foreigners, and the 2013 epidemic, associated to berries’ consumption, [26] which affected mainly Italian citizens. Excluding these two epidemics, rates recorded among foreigners are consistently higher than those among Italians in the observation period. In 2019 the standardised rates were 0.7 and 2.4 per 100,000 among Italians and foreigners respectively (*p* < 0.05).

### 3.2. Hepatitis B

Four thousand nine hundred and eighty-one hepatitis B cases were notified to SEIEVA, of which 17.0% (*n* = 849) were related to foreign citizens (Table 1). Fifty-three percent of them were from Eastern Europe (223 from Romania, 69 from Albania), 21.6% were from Africa (57 from Morocco, 38 from Nigeria) and 14.6% from Asia (47 from China) (Table 2).

**Figure 2:**
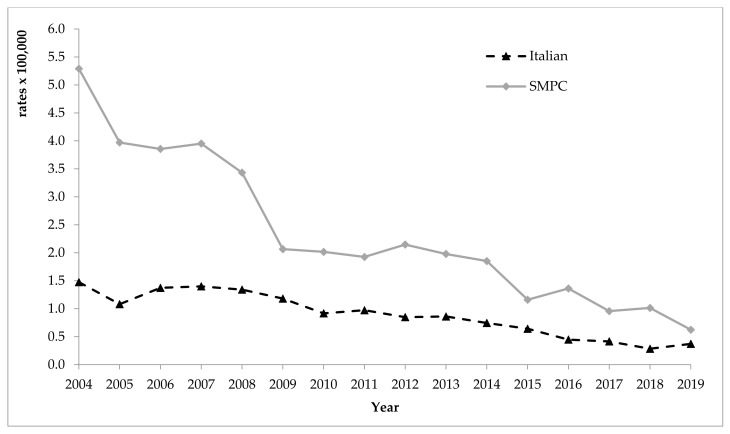
Incidence rates (standardised per 100,000) of viral acute hepatitis B in the foreign population from SMPC and among Italians, years 2004–2019. SMPC—Strong Migratory Pressure Countries.

The comparison between the observed rates among Italians and among foreigners from SMPC reveals values up to 4 times higher among foreigners from SMPC, at least until 2008 (Figure 2). Since 2009, this difference progressively decreased; the lowest distances were observed in 2015, when standardised incidence rates were 0.6 per 100,000 among Italians and 1.2 per 100,000 among migrants from SMPC, and in 2019, when standardised rates were 0.4 and 0.6 per 100,000 for Italians and foreigners, respectively (*p* > 0.05, for 2019).

### 3.3. Hepatitis C

During the period 2004–2019, 1161 cases of acute hepatitis C were notified to SEIEVA: 8.0% (*n* = 93) of them were observed among foreigners, 59.1% of which were from Eastern Europe (mainly from Romania, 20 cases) and 24.7% from Africa (mainly from Morocco, 15 cases) (Table 1 and Table 2).

**Figure 3:**
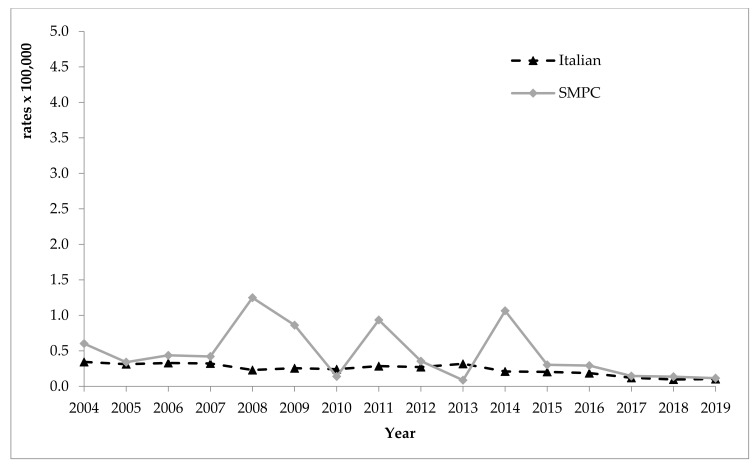
Incidence rates (standardised per 100,000) of viral acute hepatitis C in the foreign population from SMPC and among Italians, years 2004–2019. SMPC—Strong Migratory Pressure Countries.

Figure 3 shows incidence rates by year among Italians and among foreigners from SMPC. From 2004 to 2007, and later from 2015 to 2019, the two curves are almost identical. The fluctuating trend of standardised rates observed among foreigners from 2008 to 2014 is due to the small annual number of acute hepatitis C cases recorded among this population, which makes the estimates less stable. In the last 3 years, incidence rates observed in both populations are 0.1 per 100,000.

### 3.4. NonA-nonC and of Unknown Origin Hepatitis

From 2004 to 2019, out of 1322 acute hepatitis cases negative for hepatitis A, B and C (nonA-nonC) or unknown, 294 (22.2%) were observed among foreigners; almost half were from Asia (Table 1 and Table 2).

The number of hepatitis E cases diagnosed among foreigners was significant (*n* = 94–32%); almost all were observed among South Asians (mainly Bangladesh, 37 cases; India, 25 cases and Pakistan, 13 cases) (Table 3).

Eleven cases of hepatitis Delta virus were notified among migrants from SMPC, 10 of which were from Eastern Europe (Romania, Moldova and Albania) with the last case in a foreign citizen notified in 2014.

A total of 116 cases was unknown and 57 (19.4%) were classified as nonA-nonC hepatitis (HEV not tested); 25 unknown cases and 21 nonA-nonC cases were from Bangladesh, India and Pakistan (Table 3).

## 4. Discussion

In recent years, the epidemiology of viral hepatitis in Italy highlighted a general downward trend in incidence. However, the relevance of this study also relates to emerging concerns among natives that migrants may carry infectious diseases, and to the related stigma attached to them [27].

As a whole, the present study finds that the percentage of foreigners among cases notified to the SEIEVA surveillance (14.8%) is slightly higher than the estimated percentage of migrants in Italy, which is around 10% (considering documented and undocumented migrants). However, more than 86% of cases were diagnosed when subjects had resided in Italy for at least 1 year, indicating that the infection was locally acquired; on the other hand, the risk of transmission from refugees and migrants to the host population is considered to be low and mostly related to poor living conditions [28,29,30]. The distribution by country of origin of cases among migrants mirrors the presence by country of origin of migrants themselves in Italy: In fact, most cases were from geographical regions with higher presence in Italy, as Eastern Europe (mostly hepatitis B and C cases) and Africa (mostly hepatitis A) [6].

Considering infections by type, the comparison between hepatitis A rates among Italians and migrants shows a slightly higher curve for migrants from SMPC in the last two years: the excess of cases is mainly due to Moroccan nationals, mainly susceptible children, travelling to their country of origin [31]. In fact, in the period January–June 2018, two hepatitis A epidemics were reported in Europe affecting travellers returning from Morocco, with a total of 163 cases in eight countries [32]. This observation points to the need for increased vaccination efforts targeting international travellers and for sustained monitoring over the coming years.

Regarding hepatitis B, data from the SEIEVA surveillance show that foreigners from SMPC have higher incidence rates of hepatitis B, even though the distance between the curves seem to reduce over time. In fact, there is a downward trend for both population groups due to the vaccination policies in place in Italy and possibly in migrants’ countries of origin. The downward trend is less evident among Italians, probably because the peculiar vaccination strategy adopted since 1991 (vaccination of both infants and 12-year-old boys) had its effects before the observation period [20]. On the contrary, the entry of Romania and Bulgaria (where vaccination programs are in place since 1995) into the European Union in 2007 meant that a large number of these nationals entered Italy in the following years, probably having a higher vaccination coverage than new entrants in previous years [33]. Nevertheless, Eastern Europe still has the highest endemic level in Europe and one of the highest in the world [34]; as Romanians are the most numerous foreign nationals in Italy, most hepatitis B cases come from among migrants from this area.

Regarding hepatitis C, the percentage of foreigners among cases is in line or lower than the percentage of foreigners living in Italy, indicating that migrants do not seem to be at increased risk of contracting hepatitis C compared to Italians. This is confirmed by the overlapping incidence curves for the two populations.

The comparison of the incidence rates observed in the present study with those in previous years and/or in other host countries is not possible due to the lack of studies on surveillance systems which report incidence data [9]; on the contrary, there is ample information in the literature regarding migration, communicable diseases and related prevalence data. Prevalence among newly arrived migrants in Italy ranged from 11.2% to 22.7% for HBsAg and from 3.9 to 20.4 for anti-HCV [13,35] among adults, and was 2.5% (HBsAg) and 1.1 (anti-HCV) among unaccompanied children living in reception centres [36]. Considering both newly arrived and resident migrants, prevalence was 12.2% for HBsAg and from 3.3 to 6.2% for anti-HCV [12,14]; additional evidence shows that these prevalence rates are higher than the corresponding ones among Italians [16,37]. A direct comparison was carried out in two studies on pregnant women: HBsAg prevalence was significantly higher among non-Italian than among Italian women, while no anti-HCV positive woman was observed among the 711 pregnant migrants in North Eastern Italy [17]. Different findings were reported by another study on HCV conducted in Southern Italy, where a higher seroprevalence was observed among Italian women than among foreigners [18].

Concerning nonA-nonC hepatitis, most of the cases are attributable to HEV. Hepatitis E, a oro-faecal infection, is spread in Italy through two different routes: it is locally acquired, often through the consumption of raw or undercooked pork or wild boar meat. Or it is associated with travels to endemic areas [38], especially South Asia (Bangladesh, India and Pakistan) characterised by the highest HEV endemic level in the world [39]. Considering that Italian clinical centres do not always perform serological analysis for HEV, some nonA-nonC or unknown cases are possibly attributable to HEV; the geographical origin of many of the notified cases belonging to this group supports this hypothesis [40].

Concerning hepatitis Delta, acute cases seem not to be an issue, as only 11 cases were observed among migrants during the 16-year study period. On the contrary, a study conducted in 2019 in 9 clinical centres throughout Italy found an increasing anti-HDV prevalence among HBsAg positive migrants (26.4%), which is higher than the one among native hepatitis B chronic cases (4.6%) [41].

A strength of the present study is that, for the first time to our knowledge, it directly compares acute hepatitis incidence rates among foreigners and among natives in Italy. Moreover, the study has a large representativeness: while participation to SEIEVA is voluntary, it is indeed very high, covering approximately 82% of the national population, even if only 10 Italian Regions were included in the present study. Additionally, SEIEVA methodology and network remained unchanged throughout the study period; this makes the analysis of trends in incidence rates reliable, even in the presence of a possible under-notification.

On the contrary, the total number of migrants might be underestimated here, due to the possible underestimation of the number of undocumented migrants, who are all entitled to healthcare in Italy; this may have resulted in an overestimation of incidence rates among migrants.

A possible limitation of the study is that the exact time of infection is unknown and in some cases it may have occurred during the migration process and not in Italy. In any case, less than 14% of foreign cases might have been infected outside Italy (86% of foreign cases were in Italy since more than 1 year).

## 5. Conclusions

In conclusion, according to the present study, migrants have increased standardised incidence rates of acute hepatitis A and B compared to the Italian population, even though the standardised incidence rates of hepatitis B show no statistically significant difference for the year 2019. Hepatitis C rates are comparable between the two populations; as migrants are reported to have a higher prevalence of chronic hepatitis C infection [42], screening tests for HBV and HCV infections should be offered to newly arrived migrants from countries with high HBV (>2%) and HCV (>3%) prevalence, and to individuals with specific risk factors, in line with the Italian guidelines “Health assessment for migrants and asylum seekers upon arrival and while hosted in reception centres” [43]. HBsAg screening is also recommended for pregnant women, to ensure a specific prophylaxis to newborns, in case of positive mother. It is also essential to guarantee to all migrants present on the national territory levels of immunization similar to those guaranteed to the resident population, in order to safeguard individual and collective health. In particular, the active offer of hepatitis B vaccination has to be strengthened for all non-immune migrants coming from endemic areas. However, care must be taken to ensure that health interventions are culturally sensitive, i.e., designed taking into account socioeconomic factors and language and/or cultural barriers that result in poor access to vaccination, screening and link with care [44]. These measures would reduce new hepatitis B and C infections and would allow a further convergence and decrease of incidence rates in the two populations under comparison.

Many cases of hepatitis A, especially among foreigners, are associated with travel: the vaccination anti-hepatitis A for international travellers in general and for foreign citizens (especially second-generation migrant children) travelling home to endemic areas in particular should therefore be strengthened. It is also important that people who periodically return to their homelands are informed about essential preventive measures [45].

The present study is targeted to acute viral hepatitis among foreigners, but they are also reported to be at risk of other sexually transmitted infections [2]. As a consequence, a wider strategy of prevention and care, taking into account not only viral hepatitis (B, C) but also other sexual transmissible infections, is recommended by the Italian guidelines [43].

The major drivers of exposure to infectious diseases in migrants may be poor and overcrowded living conditions, socio-economic factors and linguistic and/or cultural barriers resulting in poor access and linkage to care and treatment and high-risk behaviours. Early diagnoses of various latent infections may prevent long-term sequelae, prevent onward transmission and are cost-effective for host healthcare systems [2].

Finally, it is necessary that social services, voluntary personnel and cultural mediators optimize their interventions in order to help migrants overcoming cultural and linguistic barriers which could impede their access to preventive and treatment health and social services in case of infection.

## Figures and Tables

**Table 1:** Absolute distribution and percentage of foreign citizens among acute viral hepatitis cases by type of hepatitis and by year. SEIEVA 2004–2019.

	Foreigners/N Cases (%)				
Year	Total Cases	Hepatitis A	Hepatitis B	Hepatitis C	Hepatitis nonA-nonC/Unknown
2004	153/1384 (11.0)	58/651 (8.9)	63/498 (12.6)	7/112 (6.2)	25/123 (20.3)
2005	127/969 (13.1)	46/386 (11.9)	57/374 (15.2)	4/101 (4.0)	20/108 (18.5)
2006	197/1114 (17.7)	84/407 (20.6)	69/473 (14.6)	7/109 (6.4)	37/125 (29.6)
2007	169/1040 (16.2)	56/353 (15.9)	85/487 (17.4)	6/102 (5.9)	22/98 (22.4)
2008	167/1372 (12.2)	71/742 (9.6)	68/453 (15.0)	6/74 (8.1)	22/103 (21.4)
2009	164/1303 (12.6)	84/753 (11.2)	52/390 (13.3)	8/83 (9.6)	20/77 (26.0)
2010	148/826 (17.9)	62/371 (16.7)	63/326 (19.3)	5/75 (6.7)	18/54 (33.3)
2011	153/744 (20.6)	58/244 (23.8)	61/334 (18.3)	10/88 (11.4)	24/78 (30.8)
2012	161/712 (22.6)	70/251 (27.9)	63/304 (20.7)	9/86 (10.5)	19/71 (26.8)
2013	170/1260 (13.5)	90/781 (11.5)	65/313 (20.8)	3/94 (3.2)	12/72 (16.7)
2014	146/666 (21.9))	73/281 (26.0)	54/260 (20.8)	9/69 (13.0)	10/56 (17.9)
2015	104/587 (17.7)	41/214 (19.2)	40/225 (17.8)	5/61 (8.2)	18/87 (20.7)
2016	91/544 (16.7)	39/282 (13.8)	39/168 (23.2)	3/37 (8.1)	10/57 (17.5)
2017	139/2254 (6.2)	98/2011 (4.9)	27/144 (18.7)	4/24 (16.7)	10/75 (13.3)
2018	142/590 (24.1)	101/407 (24.8)	27/109 (24.8)	3/16 (18.7)	11/58 (19.0)
2019	121/507 (23.9)	85/274 (31.0)	16/123 (13.0)	4/30 (13.3)	16/80 (20.0)
Total	2352/15,872 (14.8)	1116/8408 (13.3)	849/4981 (17.0)	93/1161 (8.0)	294/1322 (22.2)

**Table 2:** Distribution of acute viral hepatitis cases among foreign citizens by country of citizenship (level of development and geographic area), sex, age and type of hepatitis. SEIEVA 2004–2019.

Origin	Total Cases N (%)	Hepatitis A N (%)	Hepatitis B N (%)	Hepatitis C N (%)	Hepatitis nonA-nonC/Unknown N (%)
**Level of development**					
SMPC	2278 (96.9)	1061 (95.1)	834 (98,2)	92 (98.9)	291 (99.0)
HIC	74 (3.1)	55 (4.9)	15 (1,8)	1 (1.1)	3 (1.0)
**Geographic area**					
Africa	773 (32.9)	533 (47.8)	183 (21.6)	23 (24.7)	34 (11.6)
Central/South America	212 (9.0)	108 (9.7)	76 (8.9)	6 (6.4)	22 (7.5)
Asia	417 (17.7)	140 (12.5)	124 (14.6)	8 (8.6)	145 (49.3)
East Europe	886 (37.7)	288 (25.8)	453 (53.4)	55 (59.1)	90 (30.6)
West Europe	56 (2.4)	42 (3.8)	11 (1.3)	1. (1.1)	2 (0.7)
North America	6 (0.3)	4 (0.4)	1 (0.1)	0 (0.0)	1 (0.3)
Oceania	2 (0.1)	1 (0.1)	1 (0.1)	0 (0.0)	0 (0.0)
**Sex**					
Males	1426 (60.8)	610 (54.9)	544 (64,2)	56 (60.2)	216 (74.0)
Females	918 (39.2)	501 (45.1)	304 (35.8)	37 (39.8)	76 (26.1)
**Age**					
0–14	681 (29.1)	646 (58.4)	12 (1.4)	4 (4.3)	19 (6.5)
15–24	453 (19.4)	186 (16.8)	192 (22.7)	12 (12.9)	63 (21.5)
25–34	608 (26.0)	129 (11.7)	335 (39.6)	30 (32.3)	114 (38.9)
35–54	540 (23.1)	128 (11.6)	284 (33.6)	40 (43.0)	88 (30.0)
≥55	56 (2.4)	17 (1.5)	23 (2.7)	7 (7.5)	9 (3.1)
**Total**	2352 (100)	1116 (100)	849 (100)	93 (100)	294 (100)

**Table 3:** NonA-nonC or unknown hepatitis: distribution by aetiology and geographic area of country of citizenship. SEIEVA 2004–2019.

Etiologic Agent	N. of Cases (%)	Area of Origin (No. of Cases)
Delta	11 (3.7)	Eastern Europe (10), Africa (1)
E	94 (32.0)	Asia (79) Eastern Europe (6), Central/South America (5), Africa (3), West Europe (1)
nonA-nonC *	57 (19.4)	Asia (24), Eastern Europe (21), Africa (8) and Central/South America (4)
nonA-nonE **	16 (5.4)	Africa (5), Asia (5), Central/South America (4), Eastern Europe (2)
Unknown	116 (39.5)	Eastern Europe (51), Asia (37), Africa (17), Central/South America (9), West Europe (1), North America (1)
Total	294 (100)	Asia (145), Eastern Europe (90), Africa (34), Central/South America (22), West Europe (2) and North America (1)

* Cases negative for HAV, HBV, HCV and Delta and not tested for HEV. ** Cases negative for HAV, HBV, HCV, Delta and HEV.

## Data Availability

The data are not publicly available due to ethical restrictions.

## References

[1] IOM International Organization for Migration—UN Migration (2019). World Migration Report 2020.

[2] Greenaway C., Castelli F. (2019). Infectious diseases at different stages of migration: An expert review. J. Travel Med..

[3] UNHCR—The UN Refugee Agency Figure at a Glance 2020. https://www.unhcr.org/figures-at-a-glance.html.

[4] ISTAT (2020). Censimento Permanente Della Popolazione e Delle Abitazioni and Infografica Sugli Stranieri in Italia. https://www.istat.it/it/archivio/251687.

[5] Fondazione ISMU (2020). Venticinquesimo Rapporto Sulle Migrazioni 2019.

[6] Centro Studi e Ricerche IDOS (2020). Dossier Statistico Immigrazione 2020.

[7] Jefferies M., Rauff B., Rashid H., Lam T., Rafiq S. (2018). Update on global epidemiology of viral hepatitis and preventive strategies. World J. Clin. Cases.

[8] European Centre for Disease Prevention and Control (2016). Epidemiological Assessment of Hepatitis B and C among Migrants in the EU/EEA.

[9] World Health Organzation Europe (2018). Report on the Health of Refugees and Migrants in the WHO European Region: No Public Health without Refugee and Migrant Health.

[10] McNaughton A.L., Lourenço J., Bester P.A., Mokaya J., Lumley S.F., Obolski U., Forde D., Maponga T.G., Katumba K.R., Goedhals D. (2020). Hepatitis B virus seroepidemiology data for Africa: Modelling intervention strategies based on a systematic review and meta-analysis. PLoS Med..

[11] Coppola N., Alessio L., Gualdieri L., Pisaturo M., Sagnelli C., Minichini C., Di Caprio G., Starace M., Onorato L., Signoriello G. (2017). Hepatitis B virus infection in undocumented immigrants and refugees in Southern Italy: Demographic, virological, and clinical features. Infect Dis. Poverty.

[12] Cuomo G., Franconi I., Riva N., Bianchi A., Digaetano M., Santoro A., Codeluppi M., Bedini A., Guaraldi G., Mussini C. (2019). Migration and health: A retrospective study about the prevalence of HBV, HIV, HCV, tuberculosis and syphilis infections amongst newly arrived migrants screened at the Infectious Diseases Unit of Modena, Italy. J. Infect Public Health.

[13] Scotto G., Fazio V., Lo Muzio L., Coppola N. (2019). Screening for infectious diseases in newly arrived asymptomatic immigrants in southern Italy. East Mediterr. Health J..

[14] Scotto G., Armignacco O., Starnini G., Francavilla R., Foti G., Portelli V., Mazzeo M., Minerva N., Carretta V. (2016). Hepatitis C and immigration: A multicentre study. Infez. Med..

[15] European Centre for Disease Prevention and Control (2018). Hepatitis B and C Epidemiology in Selected Population Groups in the EU/EEA.

[16] Andriulli A., Stroffolini T., Mariano A., Valvano M.R., Grattagliano I., Ippolito A.M., Grossi A., Brancaccio G., Coco C., Russello M. (2018). Declining prevalence and increasing awareness of HCV infection in Italy: A population-based survey in five metropolitan areas. Eur. J. Intern Med..

[17] Lembo T., Saffioti F., Chiofalo B., Granese R., Filomia R., Grasso R., Triolo O., Raimondo G. (2017). Low prevalence of hepatitis B and hepatitis C virus serum markers in a cohort of pregnant women from Southern Italy. Dig. Liver Dis..

[18] Piffer S., Mazza A., Dell’Anna L. (2020). Serological screening for hepatitis C during pregnancy: Seroprevalence and maternal and neonatal outcomes in 45,000 pregnant women. Eur. J. Obstet. Gynecol. Reprod. Biol..

[19] Tosti M.E., Ferrigno L., Mele A., Alfonsi V., Iantosca G., Crateri S., Franca D’Angelo F., Andreozzi S. (2020). Epidemiologia Delle Epatiti Virali Acute in Italia, Bollettino SEIEVA N. https://www.epicentro.iss.it/epatite/bollettino/Bollettino-n-6-marzo-2020.pdf.

[20] Tosti M.E., Alfonsi V., Lacorte E., Mele A., Galli C., Zanetti A.R., Romanò L., SEIEVACollaborating Group Ferrigno L., Crateri S., Iantosca G. (2016). Acute Hepatitis B after the implementation of universal vaccination in Italy: Results from 22 years of surveillance (1993–2014). CID.

[21] Tosti M.E., Longhi S., De Waure C., Mele A., Franco E., Ricciardi W., Filia A. (2015). Assessment of timeliness, representativeness and quality of data reported to Italy’s national integrated surveillance system for acute viral hepatitis (SEIEVA). Public Health.

[22] Carletti P. (2009). La Salute Della Popolazione Immigrata: Metodologia di Analisi; Promozione della Salute della Popolazione Immigrata in Italia Accordo Ministero della Salute/CCM—Regione Marche (Direzione Generale Prevenzione Sanitaria, Ufficio I, n DG/PREV/I 3488/P/F 3 ad, 2007). http://www.ccm-network.it/progetto.jsp?id=node/74&idP=740.

[23] Giraudo M., Bena A., Costa G. (2017). Migrant workers in Italy: An analysis of injury risk taking into account occupational characteristics and job tenure. BMC Public Health.

[24] Leonardsson H., Hreinsson J.P., Löve A., Björnsson E.S. (2017). Hepatitis due to Epstein-Barr virus and cytomegalovirus: Clinical features and outcomes. Scand. J. Gastroenterol..

[25] Ndumbi P., Freidl G.S., Williams C.J., Mårdh O., Varela C., Avellón A., Friesema I., Vennema H., Beebeejaun K., Ngui S.L. (2018). Hepatitis A outbreak disproportionately affecting men who have sex with men (MSM) in the European Union and European Economic Area, June 2016 to May 2017. Euro Surveill..

[26] Severi E., Verhoef L., Thornton L., Guzman-Herrador B.R., Faber M., Sundqvist L., Rimhanen-Finne R., Roque-Afonso A.M., Ngui S.L., Allerberger F. (2015). Large and prolonged food-borne multistate hepatitis A outbreak in Europe associated with consumption of frozen berries, 2013 to 2014. Euro Surveill..

[27] Visalli G., Facciolà A., Carnuccio S.M., Cristiano P., D’Andrea G., Picerno I., Di Pietro A. (2020). Health conditions of migrants landed in north-eastern Sicily and perception of health risks of the resident population. Public Health.

[28] Eiset A.H., Wejse C. (2017). Review of infectious diseases in refugees and asylum seekers-current status and going forward. Public Health Rev..

[29] European Centre for Disease Prevention and Control (2015). Infectious Diseases of Specific Relevance to Newly-Arrived Migrants in the EU/EEA.

[30] Eonomopoulou A., Pavli A., Stasinopoulou P., Giannopoulos L.A., Tsiodras S. (2017). Migrant screening: Lessons learned from the migrant holding level at the Greek-Turkish borders. J. Infect Public Health.

[31] Mele A., Ferrigno L., Romanò L., Alfonsi V., D’Angelo F., Crateri S., Tosti M.E. (2021). An update on the epidemiology of hepatitis A in Italy 2015-2019. Data from the surveillance of acute viral hepatitis SEIEVA. Epidemiol. Prev..

[32] Gassowski M., Michaelis K., Wenzel J.J., Faber M., Figoni J., Mouna L., Friesema I.H., Vennema H., Avellon A., Varela C. (2018). Two concurrent outbreaks of hepatitis A highlight the risk of infection for non-immune travellers to Morocco, January to June 2018. Euro Surveill..

[33] Miglietta A., Quinten C., Lopalco P.L., Duffell E. (2018). Impact of hepatitis B vaccination on acute hepatitis B epidemiology in European Union/European Economic Area countries, 2006 to 2014. Euro Surveill..

[34] World Health Organzation (2020). Hepatitis B: Key Facts.

[35] Del Pinto R., Pietropaoli D., Russomando U., Evangelista P., Ferri C. (2018). Health status of Afro-Asian refugees in an Italian urban area: A cross-sectional monocentric study. Public Health.

[36] Marrone R., Baglio G., Bruscino G., Costanzo G., Cavani A., Mirisola C. (2020). Prevalence of latent tuberculosis infection, hepatitis B, hepatitis C, and syphilis among newly arrived unaccompanied minors living in reception centers in Rome. Int. J. Infect Dis..

[37] Boccalini S., Pellegrino E., Tiscione E., Pesavento G., Bechini A., Levi M., Rapi S., Mercurio S., Mannelli F., Peruzzi M. (2013). Sero-epidemiology of hepatitis B markers in the population of Tuscany, Central Italy, 20 years after the implementation of universal vaccination. Hum. Vaccines Immunother..

[38] Alfonsi V., Romanò L., Ciccaglione A.R., La Rosa G., Bruni R., Zanetti A., Della Libera S., Iaconelli M., Bagnarelli P., Capobianchi M.R. (2018). Hepatitis E in Italy: 5 years of national epidemiological, virological and environmental surveillance, 2012 to 2016. Euro Surveill..

[39] World Health Organzation (2020). Hepatitis E: Key Facts.

[40] Tosti M.E., Ferrigno L., Mele A., Romanò L., Fiacchini D., Bagnarelli P., Zotti C., Chironna M., Prato R., Giordani M.T. (2021). Epidemiology and surveillance of hepatitis E in Italy. Data from the SEIEVA surveillance system 2007–2019. Epidemiol. Prev..

[41] Stroffolini T., Ciancio A., Furlan C., Vinci M., Fontana R., Russello M., Colloredo G., Morisco F., Coppola N., Babudieri S. (2020). Migratory flow and hepatitis delta infection in Italy: A new challenge at the beginning of the third millennium. J. Viral Hepat..

[42] Coppola N., Alessio L., Onorato L., Sagnelli C., Macera M., Sagnelli E., Pisaturo M. (2019). Epidemiology and management of hepatitis C virus infections in immigrant populations. Infect Dis. Poverty.

[43] Tosti M.E., Marceca M., Eugeni E., D’Angelo F., Geraci S., Declich S., Della Seta M., Ferrigno L., Marrone R., Pajno C. (2020). Health assessment for migrants and asylum seekers upon arrival and while hosted in reception centres: Italian guidelines. Health Policy.

[44] Seedat F., Hargreaves S., Nellums L.B., Ouyang J., Brown M., Friedland J.S. (2018). How effective are approaches to migrant screening for infectious diseases in Europe? A systematic review. Lancet Infect Dis..

[45] Whelan J., Sonder G., van den Hoek A. (2013). Declining incidence of hepatitis A in Amsterdam (The Netherlands), 1996–2011: Second generation migrants still an important risk group for virus importation. Vaccine.

